# Angiotensin-I-converting enzyme inhibitory peptides in milk fermented by indigenous lactic acid bacteria

**DOI:** 10.14202/vetworld.2020.345-353

**Published:** 2020-02-21

**Authors:** Yuliana Tandi Rubak, Lilis Nuraida, Dyah Iswantini, Endang Prangdimurti

**Affiliations:** 1 Department of Food Science and Technology, Food Science Study Program, IPB University (Bogor Agricultural University), Bogor, Indonesia; 2 Southeast Asian Food and Agricultural Science and Technology Center, IPB University (Bogor Agricultural University), Bogor, Indonesia; 3 Department of Chemistry; and Tropical Biopharmaca Research Center, IPB University (Bogor Agricultural University), Bogor, Indonesia

**Keywords:** angiotensin-I-converting enzyme inhibitory peptides, fermented milk, indigenous lactic acid bacteria, *Lactobacillus kefiri*

## Abstract

**Background and Aim:**

Fermented milk can be used to produce antihypertensive peptides. Lactic acid bacteria (LAB) with its proteolytic system hydrolyze milk protein during fermentation to produce several peptides, which include antihypertensive bioactive peptides. This study aimed to investigate the ability of indigenous LAB for the production of angiotensin-I-converting enzyme inhibitory (ACE-I) peptides in fermented milk and to characterize the ACEI peptides.

**Materials and Methods:**

Reconstituted milk (11%) inoculated with ten LAB isolates, and then incubated at 37°C until it reaches pH 4.6. The evaluation was carried out for LAB count, lactic acid concentration, peptide content, and ACE-I activity. The low molecular weight (MW) peptides (<3 kDa) were identified using Nano LC Ultimate 3000 series system Tandem Q Exactive Plus Orbitrap high-resolution mass spectrometry.

**Results:**

The result showed that the ten LAB isolates were able to produce ACE-I in fermented milk with the activities in the range of 22.78±2.55-57.36±5.40%. The activity of ACE-I above 50% produced by *Lactobacillus delbrueckii* BD7, *Lactococcus lactis* ssp. *lactis* BD17, and *Lactobacillus kefiri* YK4 and JK17, with the highest activity of ACE-I produced by *L. kefiri* YK4 (IC_50_ 0.261 mg/mL) and *L. kefiri* JK17 (IC_50_ 0.308 mg/mL). Results of peptide identification showed that *L. kefiri* YK 4 could release as many as 1329, while *L. kefiri* JK 17 could release 174 peptides. The peptides produced were 95% derived from casein. The other peptides were from ά-lactalbumin, β-lactoglobulin, and serum amyloid A. The peptides produced consisted of 6-19 amino acid residues, with MWs of 634-2079 Dalton and detected at 317-1093 m/z. A total of 30 peptides have been recognized based on literature searches as ACE-I peptides (sequence similarity: 100%).

**Conclusion:**

*L. kefiri* YK4 and JK17 are the potential to be used as starter cultures to produce the bioactive peptide as ACE-I in fermented milk.

## Introduction

Fermented milk has been proven to improve and maintain human health. It relates to the biological activities found in fermented milk, such as antioxidant, immunomodulatory, anti-inflammatory, antibacterial, anticancer, and antihypertensive activities [1-4]. Antihypertensive activity of fermented milk is one of the interesting areas due to the increases of hypertension cases and side effects caused by the use of synthetic drugs such as captopril, lisinopril, and enalapril [5]. Angiotensin-I-converting enzyme (ACE, EC 33.4.15.1, CD143) plays a key role in the blood pressure regulation system. ACE converts inactive decapeptide Angiotensin I to the potent vasoconstrictor, i.e., the octapeptide Angiotensin II and also inactivates bradykinin, a vasodilator [6,7]. ACE is one of the effective targets to reduce blood pressure. ACE-inhibitory (ACE-I) peptides can be isolated from fermented milk and are a safe, natural source to be used in the treatment of hypertension. Milk protein is a precursor of bioactive peptides [8-10], but most of the peptides in milk are inactive that requiring hydrolysis to release them. Fermentation of milk involving lactic acid bacteria (LAB) such as in yogurt, cheese, and other fermented milk is a method known to hydrolyze proteins and release bioactive peptides from the primary protein structure [11,12]. The proteolytic system makes LAB as a prospective producer of bioactive antihypertensive peptides in fermented food products [13,14]. Proteolytic activity of LAB varies between species and even strains. Therefore, it is necessary to find a LAB with high proteolytic activity to produce bioactive antihypertensive peptide. The LAB which has been reported by several researchers as being able to produce bioactive antihypertensive peptides in fermented milk among others are *Lactobacillus helveticus* [13,15], *Lactobacillus plantarum*, *Lactobacillus pentosus*, *Pediococcus acidilactici*, *Pediococcus pentosaceus*, *Lactobacillus delbrueckii* ssp. *bulgaricus*, and *Lactococcus ­lactis* [16,17]. *L. helveticus* has been widely used in dairy products to generate ACE-I peptides [18]. Calpis, which is a soft drink manufactured in Japan, is produced from milk fermented by a mixture of *L. helveticus* CP790 and *Saccharomyces cerevisiae* [19]. Two bioactive peptides with ACE-I activity have been identified in calpis, i.e., Val-Pro-Pro (VPP) and Ile-Pro-Pro (IPP). VPP and IPP peptides have the same structure as captopril and enalapril [20] commonly used in hypertension therapy [21].

Screening of LAB capable of producing bioactive peptides as ACE-I and characterization of the resulted peptides are essential to obtain isolates that can be used as starter cultures to produce fermented milk containing antihypertensive peptides. The availability of indigenous LAB with the ability to produce specific peptides as a starter culture will also contribute to the diversification of fermented milk products as a functional food.

This study aimed to explore the capacity of ten indigenous LAB that previously has been isolated from tempe, kefir, and breast milk to generate ACE-I peptides and to characterize the ACE-I peptides generated.

## Materials and Methods

### Ethical approval

No animals were used in the present study, so there was no requirement of ethical approval.

### LAB

The ten LAB were *P. pentosaceus* 1 W2SR04, *L. plantarum* 1 W22408, *Lactobacillus rhamnosus* R2, *Lactobacillus* R7F, *L. delbrueckii* BD7, *L. lactis* ssp. *lactis* BD17, *Lactobacillus* *fermentum* R6, S206, and *Lactobacillus kefiri* YK4 and JK17 that previously have been isolated from tempe, kefir, and breast milk were obtained from Food Microbiology Laboratory of the Southeast Asian Food and Agricultural Science and Technology (SEAFAST) Center, IPB University, Bogor, Indonesia.

### Fermentation of milk

The LAB isolates were refreshed by growing them in De Man, Rogosa, and Sharpe broth (Oxoid, USA) and incubated at 37°C for 24 h. To prepare inoculum for milk fermentation, the isolates were inoculated into 11% skim milk and incubated for 24 h at 37°C. The starter culture (2%) was inoculated into pasteurized (95°C for 10 min) reconstituted skim milk (11% NZWP Ltd., NZ) and then incubated at 37°C to reach pH 4.6 (pH 700 Eutech). When the pH has been reached, the LAB count and titratable acidity were analyzed. The fermentation process was then stopped by heating at 75°C for 1 min. The fermented milk was then centrifuged (Hettich, Zentrifugen, Mikro 22R) at 6000× *g* 10 min at 4°C. For the analysis of soluble protein, peptide, and ACE-I activity, the supernatant was collected. The titratable acidity was determined by the titration, while the LAB was enumerated on De Man, Rogosa, and Sharpe Agar (Oxoid, USA).

### Determination of peptide content

The o-phthaldialdehyde (OPA) method was used to measure peptide content [22]. A total of 50 µL samples were mixed with 2 mL of OPA reagents (consisting of 25 mL of 100 mM of sodium tetraborate, 2.5 mL of 20% (w/w) of sodium dodecyl sulfate, and 1.1 mL of OPA solution, mixed with 21.4 mL of dH_2_O). The OPA solution was prepared by dissolving 40 mg of OPA (Sigma, USA) in 1 mL of methanol+100 mL of ß-mercaptoethanol (Sigma, USA). The sample and OPA reagent were quickly mixed with and then incubated for 2 min, then the sample absorbance was measured at 340 nm (UV-VIS-1240, Shimadzu, Kyoto, JPN). The peptide content was quantified using the tryptone casein (Merck, USA) standard curve.

### Determination of soluble protein content

Protein content was determined using the Bradford method [23]. A total of 10 µL samples were mixed with 250 µL Bradford reagent and then incubated for 5 min. The absorbance of the sample was measured at 595 nm (Biorad, iMark, Microplate Reader, JPN). The protein content of the sample was calculated using a standard curve of bovine serum albumin (Sigma, USA).

### Determination of ACE-I activity

The determination of ACE-I activity was performed *in vitro* based on the method of Chusman and Cheung [24]. Hippuryl-L-Histidyl-L-Leucine (HHL, Sigma, USA) was used as the enzyme-substrate. A 50 µL of the substrate (50 mM HHL in 0.1 M sodium borate buffer containing 0.3 M NaCl at pH 8.3) was added into 50 µL sample and incubated at 37°C for 5 min. To initiate the reaction, 50 µL of 0.1 U/mL ACE (Sigma, USA) solution was added, and the mixture was incubated at 37°C for 5 min. The reaction was stopped by adding 250 µL 1 M HCl 1 M. The resulted in hippuric acid (HA) was extracted with 1.5 mL ethyl acetate and centrifuged at 2000× *g* for 5 min. An aliquot (0.8 mL) of the ethyl acetate layer was transferred to a clean tube and evaporated at 85°C for 60 min. Distilled water (4 mL) was then added to dissolve the HA in the tube, and the amount of HA formed was measured by measuring optical density at 228 nm (UV-2800, Hitachi, JPN) The extent of inhibition was calculated as 100% ([B-A]/B) where A is the optical density in the presence of ACE and ACE-I component, B is the optical density without ACE-I component.

### IC_50_ value

The value of IC_50_ was calculated based on the equation obtained from the curve of ACE inhibition as a function of different concentrations of peptide. The value of IC_50_ is defined as the amount of peptide required to inhibit 50% of ACE activity.

### Characterization of peptides from fermented milk

#### Partial purification

The supernatant of fermented milk that has the highest ACE-I activity was pipetted into ultrafiltration centrifuge tubes molecular weight (MW) cutoff of 3 kDa (Merck, Amicon Ultra-4 mL, Centrifugal Filters, IRL). We collected two fractions (MW <3 kDa and MW >3 kDa) and assayed their ACE-I activity.

#### Identification of peptides by mass spectrometry (MS)

The characterization of peptide (MWs and amino acid sequences) was carried out in Advanced Research Laboratory, IPB University (Bogor Agricultural University) based on the method of Daliri *et al*. [25] using Nano Liquid Chromatography (LC) Ultimate 3000 series system Tandem Q Exactive Plus Orbitrap high-resolution MS (Thermo Scientific, GER). Fractions <3 kDa (5 µL) of the samples were injected into the LC-nano MS system. The samples were trapped on a trap column (Thermo Scientific, 164649, 30 µm×5 mm) and washed for 6 min with gradient with 98% Solvent A (water/acetonitrile [98:2, v/v], 0.1% formic acid) and 2% Solvent B (Water/acetonitrile [2:98, v/v], 0.1% formic acid) at a flow rate of 5 µL/min. The peptides were separated on a capillary column (PepMap RSLC-C18, 75-µm×150 mm, 3.5 µm particle size, 100 pore size, part number ES 800, Thermo Scientific) at a flow rate of 300 nL/min with gradient at 2-35% Solvent B over 30 min, then from 35% to 90% over 10 min, followed by 90% Solvent B for 5 min, and finally 5% Solvent B for 15 min. Electrospray was performed at an ion spray voltage of 3500 eV. The range of m/z values was 200-2000. The peptides were analyzed using Proteomic Discoverer 2.2 software.

### Statistical analysis

All analyses were carried out in triplicate and expressed as mean±standard deviation. The data obtained were analyzed by Analysis of Variance. The differences between means were assessed using the Duncan test and were considered significant when p≤0.05. Statistical analysis was performed using SPSS version 16 software (IBM Corp., NY, USA).

## Results

### Growth of LAB in milk

Table-1 shows the growth of LAB, titratable acidity, and fermentation time required by 10 LAB to reach pH 4.5. Changes in pH due to the accumulation of lactic acid as a result of lactose metabolism during fermentation ranged from 0.77±0.06 to 0.91±0.06%. The fermentation time required to reach pH 4.6 by 10 LAB was 24-48 h. The lactic acid counts ranged from 9.11±0.22 to 9.62±0.28 log CFU/mL. The soluble protein content in the sample ranged from 0.215±0.01 to 0.395±0.00 mg/mL (Table-1).

**Table-1:** Fermentation time, titratable acidity, viable count of LAB, and soluble protein content in fermented milk after pH 4.6 were reached.

Starter culture	Fermentation time (h)	Titratable acidity (%)	Viable LAB (log CFU/mL)	Soluble protein content (mg/mL)
*Lactobacillus rhamnosus* R2	32	0.77±0.06	9.46±0.36	0.215±0.01
*Lactobacillus kefiri* JK17	32	0.84±0.05	9.62±0.40	0.395±0.00
*Lactobacillus delbrueckii* BD7	48	0.83±0.04	9.41±0.25	0.383±0.01
*Lactobacillus fermentum* S206	48	0.87±0.05	9.33±0.37	0.277±0.01
*Lactobacillus kefiri* YK4	32	0.91±0.06	9.62±0.28	0.343±0.00
*Lactobacillus* R7F	40	0.84±0.02	9.11±0.22	0.386±0.02
*Lactobacillus fermentum* R6	24	0.80±0.06	9.49±0.26	0.384±0.01
*Lactobacillus plantarum* 1W22408	32	0.79±0.04	9.21±0.41	0.277±0.01
*Lactococcus lactis* ssp. *lactis* BD17	40	0.82±0.08	9.25±0.42	0.361±0.01

LAB=Lactic acid bacteria

### ACE-I activity

The percentages of ACE-I activity, peptide content, and inhibitory efficiency ratio (IER) values in milk fermented by 10 LAB cultures are shown in Table-2. ACE-I activity ranged from 22.78±2.55% to 57.36±5.40% in fermented milk. ACE-I activity above 50% was found in milk fermented by *L. delbrueckii* BD7, *L. lactis* ssp. *lactis* BD17, and *L. kefiri* YK4 and JK17. The highest percentage of ACE-I was obtained in milk fermented by *L. kefiri* YK4 and *L. kefiri* JK17, but it was not significantly different (>0.05) with milk fermented by *L. delbrueckii* BD7 and *L. lactis* ssp. *lactis* BD17. As a positive control, captopril was used which generated ACE-I activity of 87.5±1.05%.

**Table-2:** ACE-inhibitory, peptide content, and IER in milk fermented by ten LAB cultures after reaching pH 4.6 incubated at 37°C.

Starter culture	% inhibition of ACE	Peptide content (mg/mL)	IER (% per mg/mL)
*Lactobacillus rhamnosus* R2	22.78±2.55^e^	4.012±0.20^a^	5.68±0.62^e^
*Lactobacillus kefiri* JK17	56.53±1.53^a^	3.976±0.17^a^	14.23±0.53^a^
*Lactobacillus delbrueckii* BD7	50.97±2.46^ab^	3.727±0.27^ab^	13.79±1.60^ab^
*Lactobacillus fermentum* S206	40.83±3.55^cd^	3.676±0.21^abc^	11.12±0.84^c^
*Lactobacillus kefiri* YK4	57.36±5.40^a^	3.540±0.07^abcd^	16.24±1.85^a^
*Lactobacillus* R7F	27.78±2.89^e^	3.322±0.21^bcd^	8.44±1.32^d^
*Lactobacillus fermentum* R6	44.86±1.87^bc^	3.266±0.22^bcd^	13.81±1.13^ab^
*Lactobacillus plantarum* 1W22408	46.94±3.42^bc^	3.219±0.29^de^	14.81±0.70^a^
*Lactococcus lactis* ssp. *lactis* BD17	52.22±4.86^ab^	3.211±0.26^de^	16.33±1.72^a^
*Pediococcus pentosaceus* 1 W2SR04	35.14±3.27^d^	3.116±0.08^e^	11.27±0.99^c^
Captopril	87.5±1.05		

ACE=Angiotensin-I-converting enzyme, IER=Inhibitory efficiency ratio

Different superscripts in the same column indicates significant (p<0.05) between samples

### IC_50_ value

The IC_50_ value was measured in fermented milk with the highest activity of ACE-I (fermented milk of *L. kefiri* JK17 and YK4). Captopril was also measured as a control (Table-3). IC_50_ values reflect the concentration of peptide required to inhibit 50% ACE.

**Table-3:** IC_50_ value of Captopril and milk fermented by *Lactobacillus kefiri* YK4 and *Lactobacillus kefiri* JK17.

IC_50_ value (mg/mL)		

Fermented milk		Control
*Lactobacillus kefiri* YK4	*Lactobacillus kefiri* JK17	Captopril
0.261±3.94^a^	0.308±8.16^b^	0.008±0.23^c^

Different superscripts in the same row indicates significant (p < 0.05) between samples

### Partial purification

Results of filtration using a membrane with MW cutoff <3 kDa yielded two fractions, i.e., >3 kDa and 3 kDa with ACE-I activity as shown in Table-4. The whole supernatant of *L. kefiri* YK4 achieved the highest ACE-I activity (57.36±5.40%) but it was not significantly different (>0.05) from the <3 kDa fraction (55.45±1.97%). Meanwhile, the ACE-activity of the whole supernatant from milk fermented by *L. kefiri* JK17 (56.53±1.53%) was significantly higher than both fractions of >3 kDa (24.03±1.64%) and <3 kDa (52.28±4.09%).

**Table-4:** ACE-I activity of supernatant, fractions of >3 kDa and <3 kDa in milk fermented by *Lactobacillus kefiri* YK4 and *Lactobacillus kefiri* JK17.

Starter culture	ACE-I activity (%)		

	Supernatant	>3 kDa	<3 kDa
*Lactobacillus kefir* YK4	57.36±5.40^a^	31.89±2.08^b^	55.45±1.97^a^
*Lactobacillus kefir* i JK17	56.53±1.53^a^	24.03±1.64^b^	52.28±4.09^c^

ACE-I=Angiotensin-I-converting enzyme inhibitor

Different superscripts in the same row indicates significant (p < 0.05) between samples

### Identification of ACE-I peptides

Peptide profile in fermented milk (<3 kDa) by *L. kefiri* YK4 and *L. kefiri* JK17 is presented in Table-5. Identification of peptides in the fraction <3 kDa showed that the majority of peptides had m/z values range of 317-1093 with MWs of 634-2079 Dalton, and the number of residues was 6-20 amino acids. The peptides produced from both samples were derived from the hydrolysis of the casein parent protein (αS1-casein, αS2-casein, β-casein, and κ-casein), ά- lactalbumin, β-lactoglobulin, and serum amyloid A.

**Table-5:** Fermented milk peptide profile (<3 kDa) of *Lactobacillus kefiri* YK4 and *Lactobacillus kefiri* JK17.

Parent protein	Peptide total	Range of precursor m/z	Range of precursor MW	Amino acid residue	Dominant peptide	LAB
αS1-casein	92	374-971	747-1941	6-18	FSDIPNPIGSE	YK 4
	76	438-1040	876-2079	7-19	FSDIPNPIGSENSEKTTMP, FVAPFPEVFGKEK	JK 17
αS2-casein	42	325-882	810-1763	6-15	QGPIVLNPWDQVKR	YK 4
	33	424-680	848-2040	7-17	LYQGPIVLNPWDQVKRN	JK 17
β-casein	774	327-1093	979-2185	6-18	MPFPKYPVEPF	YK 4
	38	317-1022	634-2044	6-19	MPFPKYPVEP	JK 17
κ-casein	362	300-954	841-2038	6-18	ARHPHPHLSF	YK 4
	19	324-889	924-1777	7-18	FMAIPPKKNQD, VRSPAQILQ	JK 17
ά- lactalbumin	21	400-750	808-1499	7-13	KVGINYWLAH, FHTSGYDTQ	YK 4
	3	563	1126	10	FHTSGYDTQA	JK 17
β-lactoglobulin	17	322-727	775-1453	7-13	KALPMHIR	YK 4
	5	382-622	802-1243	7-11		JK 17
serum amyloid A	21	242-727	998-1753	8-15	EWGRSGKDPNHFRPA	YK 4

A=Alanine (Ala), C=Cysteine (Cys), D=Aspartic acid (Asp), E=Glutamic acid (Glu), F=Phenylalanine (Phe), G=Glycine (Gly), H=Histidine (His), I=Isoleucine (Ile), K=Lysine (Lys), L=Leucine (Leu), M=Methionine (Met), N=Asparagine (Asn), P=Proline (Pro), Q=Glutamine (Gln), R=Arginine (Arg), S=Serine (Ser), T=Threonine (Thr), V=Valine (Val), W=Tryptophan (Trp), Y=Tyrosine (Tyr)

A total of 1329 peptides were found in the fermented milk of *L. kefiri* YK4 starter culture (the whole data are not presented). As much as, 95.6% of resulted peptides were originated from casein with the proportion of 58.24% β-casein, 27.24% κ-casein, 6.92% αS1-casein, and 3.16% αS2-casein. Other peptides were 1.58% α-lactalbumin, 1.28% β-lactoglobulin, and 21.58% serum amyloid A. The dominant peptides derived from β-casein were identified as ARHPHPHLSF and MPFPKYPVEPF (Table-5). In fermented milk supernatant of *L. kefiri* JK17, 174 peptides were identified (the whole data are not shown). The peptides found were 95% derived from casein with the proportion of 43% αS1-casein, 21.8% β-casein, 19% αS2-casein, and 10.9% κ-casein. Other peptides were 1.7% ά-lactalbumin and 2.9% β-lactoglobulin. The dominant peptides found were identified as FSDIPNPIGSENSEKTTMP, FVA PFPEVFGKEK, LYQGPIVLNPWDQVKRN, and MPFPKYPVEP (Table-5).

**Figure-1:**
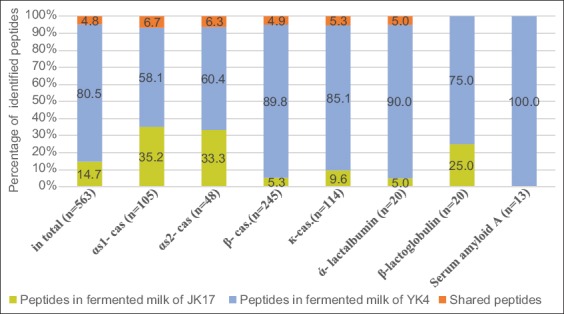
Distribution of identified peptides in fermented milk of *Lactobacillus kefiri* YK4 and JK17 in total peptides and for all parent protein, i.e., αS1-, αS2, β- and κ-casein, ά-lactalbumin, β-lactoglobulin, and amyloid A.

The distribution of peptides is presented in Figure-1. Based on the type of peptide, 480 different peptides were found in samples with starter culture *L. kefiri* YK4 and 110 peptides in samples with starter culture *L. kefiri* JK17. A total of 27 similar peptides were found in both samples.

The identification of peptides with ACE-I activity was performed by searching the peptides that have been reported in the literature. Table-6 shows the peptides that were reported to have ACE-I activity. The ACE-I peptides presented in Table-6 are only homologous peptides (sequence similarity: 100%) to the peptides that have been reported to have ACE-I activity [26-43].

**Table-6:** ACE-I peptides produced in fermented milk using *Lactobacillus kefiri* YK4 and JK17 as starter culture compared to ACE-I peptides as reported in the literature.

Parent protein	Sequence peptide	m/z [Da]	MH+ [Da]	Charge	ACE-I peptides based on literature	

					Sequence	References
K-casein	ARHPHPHLSFM	443.89	1329.66	3	ARHPHPHLSFM	[26]
β-casein	DELQDKIHPF	621.31	1241.61	2	DELQDKIHPF	[27]
β-casein	DKIHPF	378.71	756.41	2	DKIHPF	[28]
β-casein	DKIHPFAQ	478.25	955.50	2	DKIHPFAQ	[29]
β-casein	ELQDKIHPF	563.80	1126.59	2	ELQDKIHPF	[28]
β-casein	EMPFPKYPVEPF	740.86	1480.72	2	EMPFPKYPVEPF	[30]
αS1-casein	FVAPFPEV	453.24	905.48	2	FVAPFPEV	[31]
					FVAPFPEV	[32]
αS1-casein	FVAPFPEVFG	555.29	1109.57	2	FVAPFPEVFG	[30]
					FVAPFPEVFG	[32]
αS1-casein	FVAPFPEVFGK	619.33	1237.66	2	FVAPFPEVFGK	[33]
αS1-casein	FVAPFPEVFGKE	683.85	1366.70	2	FVAPFPEVFGKE	[31]
β-casein	GPVRGPFPI	470.27	939.54	2	GPVRGPFPI	[34]
αS1-casein	IGSENSEKTTMP	647.30	1293.60	2	IGSENSEKTTMP	[35]
β-casein	LGPVRGPFP	470.27	939.54	2	LGPVRGPFP	[36]
β- casein	LVYPFPGPIHNSLPQ	839.95	1678.89	2	LVYPFPGPIHNSLPQ	[30]
β- casein	LVYPFPGPIHNSLPQN	896.97	1792.93	2	LVYPFPGPIHNSLPQN	[3]
β-casein	LYQEPVLGPVRGPFPIIV	997.58	1994.14	2	LYQEPVLGPVRGPFPIIV	[31]
β-casein	MPFPKYPVEP	602.80	1204.60	2	MPFPKYPVEP	[35]
β-casein	MPFPKYPVEPF	676.34	1351.67	2	MPFPKYPVEPF	[37]
β-casein	QEPVLGPVRGPFP	696.88	1392.76	2	QEPVLGPVRGPFP	[36]
					QEPVLGPVRGPFP	[38]
β-casein	QEPVLGPVRGPFPIIV	859.50	1718.00	2	QEPVLGPVRGPFPIIV	[39]
					QEPVLGPVRGPFPIIV	[40]
β-casein	RDMPIQAF	489.25	977.49	2	RDMPIQAF	[41]
αS1-casein	RPKHPIKH	338.21	1012.61	3	RPKHPIKH	[29]
β-casein	SQSKVLPVPQ	541.81	1082.62	2	SQSKVLPVPQ	[35]
αS1-casein	VAPFPEVFGK	545.80	1090.59	2	VAPFPEVFGK	[38]
αS1-casein	VAPFPEVFGKE	610.32	1219.63	2	VAPFPEVFGKE	[32]
β-casein	VLGPVRGPFP	519.81	1038.61	2	VLGPVRGPFP	[42]
β-casein	YPFPGPIPN	501.26	1001.51	2	YPFPGPIPN	[43]
β-casein	YQEPVLGPVR	579.32	1157.63	2	YQEPVLGPVR	[43]
β-casein	YQEPVLGPVRGPFPI	556.98	1668.91	3	YQEPVLGPVRGPFPI	[33]
β-casein	YQEPVLGPVRGPFPIIV	941.03	1881.06	2	YQEPVLGPVRGPFPIIV	[31]
					YQEPVLGPVRGPFPIIV	[33]

A=Alanine (Ala), C=Cysteine (Cys), D=Aspartic acid (Asp), E=Glutamic acid (Glu), F=Phenylalanine (Phe), G=Glycine (Gly), H=Histidine (His), I=Isoleucine (Ile), K=Lysine (Lys), L=Leucine (Leu), M=Methionine (Met), N=Asparagine (Asn), P=Proline (Pro), Q=Glutamine (Gln), R=Arginine (Arg), S=Serine (Ser), T=Threonine (Thr), V=Valine (Val), W=Tryptophan (Trp), Y=Tyrosine (Tyr)

## Discussion

During milk fermentation by LAB, the bacteria ferment lactose into organic acid, mainly lactic acid, and bring the pH down. The time to reach pH 4.6 varied between LAB showing the difference in the rate of growth. Short fermentation time (24 h) was observed in milk fermented by *P. pentosaceus* 1 W2SR04 and *L. fermentum* R6, while the longest fermentation time (48 hours) occurred in milk fermented by *L. delbrueckii* BD7 and *L. fermentum* S206. A previous study by Chen *et al*. [13] report that the time to reach a pH of 4.6 by 37 *Lactobacillus* varied from 7 to 42.4 h. Meanwhile, *L. delbrueckii* QS306 required incubation time of 48 h to reach a pH of less than 4.7 [44].

All LAB isolates grew well during fermentation, with a population of more than 9 log CFU/mL (Table-1). A similar result of a viable count of LAB in fermented milk was reported by Elkhtab *et al*. [45] that of eight LAB dominated by *Lactobacillus* in fermented milk reached the population of 9.5 log CFU/mL after 72 h incubation. Similarly, in the fermented milk of *L. delbrueckii* ssp. *bulgaricus* 1466, the population reached >8 log CFU/mL when the fermented milk reaches a pH 4.5 [46].

The LAB also hydrolyzes protein in milk into peptides and amino acids to support their growth. Protein hydrolysis by LAB produces bioactive peptides acting as ACE-Is. The activity of ACE-I produced in fermented milk varies between LAB fermenting the milk [21,42] and is strain-specific [47]. *L. delbrueckii* and *L. lactis* isolates that produce high ACE-I activity in this study were similar to the previous report by Wu *et al*. [48] that *L. delbrueckii* strain QS306 could produce 75.58±1.69% of ACE-I activity in fermented milk and *L. lactis* ADP strains Q1, Q2, and Q5 could produce 90-98% of ACE-I activity after 48 h fermentation [49]. Meanwhile, the ACE-I activity in milk fermented by *L. kefiri* that isolated from kefir has not been reported.

ACE-I activity produced in fermented milk is associated with LAB’s proteolytic activity. LAB’s proteolytic activity generates several peptides. The peptides produced in this study ranged from 3.116±0.08 to 4.012±0.20 mg/mL. The highest peptide content was produced in milk fermented by *L. rhamnosus* R2 and *L. kefiri* JK17, but it was not significantly different from milk fermented by *L. delbrueckii* BD7, *L. kefiri* YK4, and *L. fermentum* S206. A high proteolytic activity has been reported in *Lactobacillus* species. The report has led to the widespread use of *Lactobacillus* species for the production of antihypertensive peptides (ACE-Is) [41,50] in the fermented milk industry, such as sour milk and yogurt.

The peptide content, however, does not always correlate with the activity of ACE-I. The results obtained in this research show a poor correlation between peptide content and ACE-I activity (Table-2). Similar results were also obtained by Chen *et al*. [13], where the percentage of ACE-I showed a poor correlation with the amount of free amino acid in fermented milk of *L. helveticus*. Similar results were also observed in milk fermented by *Leuconostoc mesenteroides* 356 [3,51]. Each LAB will produce different pieces of the peptide with its specific proteolytic system. Bioactive peptides with the potential of ACE-I are specific peptides. The peptide type determines the activity of ACE-Is. However, high proteolytic activity is needed to produce several bioactive peptides in fermented milk, including the bioactive ACE-I peptide.

The value of IER ranged from 5.68±0.62 to 16.33±1.72. The value of IER represents the effectiveness of peptide in inhibiting ACE activity. The value of IER is obtained by dividing the percentage of ACE-I activity by peptide content. High IER values indicate inhibitory efficiency against ACE. The highest IER value was obtained in milk fermented by *L. lactis* ssp. *lactis* BD17, but the difference was not significant (>0.05) than milk fermented by *L. kefiri* YK4, *L. kefiri* JK17, and *L. plantarum* 1 W22408.

Fermented milk of *L. kefiri* YK4 had a lower IC_50_ value than fermented milk of *L. kefiri* JK 17. IC_50_ values obtained from both samples were still lower with that of reported by Qian *et al*. [52] who used *L. delbrueckii* ssp. *bulgaricus* LB to ferment milk and resulted in the IC_50_ values of 67.71±7.62 mg/mL; Moslehishad *et al*. [53] used *L. rhamnosus* PTCC 1637 with the IC_50_ value of 3.947±0.029 mg/mL; and Chen *et al*. [54] in koumiss with IC_50_ of 52.47±2.87 mg/mL.

After partial purification using membrane filtration 3 kDa, the activity ACE-I of <3 kDa fraction of *L. kefiri* YK4 was not significantly different from the whole extract, but significantly higher than the fraction of >3 kDa. This finding indicated that the active peptides were mainly at <3 kDa. The 3 kDa filtration is a widely used and effective way of obtaining and identifying ACE-I peptides, as ACE-I activity is produced mainly by peptides with <3 kDa fractions [26,42,55]. However, the whole supernatant of *L. kefiri* JK17 was significantly higher than its <3 kDa fraction. This result is similar to the results of Chen *et al*. [54] that koumiss fermented milk supernatant had a higher ACE-I activity compared to the <3 kDa fraction.

The dominant proteins hydrolyzed by *L. kefiri* YK4 were β-casein and αs1-casein by *L. kefiri* JK 17 (Table-5). This finding explains the difference between the two strains in the proteolytic system. The hydrolysis of non-casein proteins (ά-lactalbumin, β-lactoglobulin, and serum amyloid A) also showed that both isolates had high proteolytic activity. Some LAB such as *Bifidobacterium* and others are known to produce peptides only from casein. Peptides that have ACE-I activity (Table-6) are generally originated from β-casein, αS1-casein, and ĸ-casein. *L. kefiri* YK4 hydrolyzed 362 peptides from ĸ-casein in fermented milk. ĸ-casein is glycomacropeptides consisting of glycine chains reported in several studies that are heavily hydrolysis-resistant [56]. This resistance was related to the presence of hydrophilic amino acids and negative charges.

ACE-I activity was related to the structure of the peptide [57]. A total of 21 ACE-I peptides obtained from the present research (Table-6) are peptides that have amino acid proline residues in the C-terminal chain. Residues of tripeptide amino acids, such as proline in the C-terminal chain, play a significant part in binding to the active side ACE [57]. In addition to proline, phenylalanine amino acid residue was also identified in 19 ACE-I peptides in the C-terminal chain. Residues of lysine amino acid were also found in five peptides of ACE-I (Table-6).

In this study (Table-6), the peptide ACE-I has a residue of 6-16 amino acids with an MW of <2 kDa. Some researchers have previously reported that ACE-I bioactive peptides are generally short peptides with residues of 2-6 amino acids [58,59] or longer peptides with 9-16 amino acids [4,60] even up to 20 amino acid residues [30,61].

## Conclusion

The ten LAB isolates produced ACE-I activity in fermented milk. The activity of ACE-I above 50% was obtained in milk fermented by *L. delbrueckii* BD7, *L. lactis* ssp. *lactis* BD17, and *L. kefiri* YK4 and JK17. The highest activity of ACE-I was found in milk fermented by *L. kefiri* YK4 (IC_50_ of 0.261 mg/mL) and *L. kefiri* JK17 (IC_50_ of 0.308 mg/mL). Results of peptide identification in the <3 kDa fraction showed that *L. kefiri* YK4 and *L. kefiri* JK17 could release peptides. The peptides produced were 95% derived from casein. A total of 30 peptides obtained in the present study have been recognized based on the literature searches as ACE-I peptides. *L. kefiri* YK4 and JK17 have the potential to be used as starter cultures in the production of bioactive ACE-I peptides. The two isolates could be used to support the strategies for promoting fermented milk products as a source of bioactive peptides that will benefit to health.

## Authors’ Contributions

LN, YTR, DI, and EP designed the research and experimental protocol. YTR carried out the research and data analyses. LN approved fermentation and analysis of the lactic acid bacteria. DI and EP approved analyses of angiotensin-converting enzyme and identification of peptides. YTR wrote the manuscript and LN corrected the manuscript. All the authors have read and approved the final version.
